# Impact of systemic vascular risk factors on the choriocapillaris using optical coherence tomography angiography in patients with systemic hypertension

**DOI:** 10.1038/s41598-019-41917-4

**Published:** 2019-04-09

**Authors:** Jacqueline Chua, Calvin Woon Loong Chin, Bingyao Tan, Si Han Wong, Kavya Devarajan, Thu-Thao Le, Marcus Ang, Tien Yin Wong, Leopold Schmetterer

**Affiliations:** 1 Singapore Eye Research Institute, Singapore National Eye Centre, Singapore, Singapore; 2 Academic Clinical Program, Duke-NUS Medical School, Singapore, Singapore; 3 National Heart Research Institute Singapore, National Heart Centre Singapore, Singapore, Singapore; 4 Department of Ophthalmology, Yong Loo Lin School of Medicine, National University of Singapore and National University Health System, Singapore, Singapore; 5 Department of Ophthalmology, Lee Kong Chian School of Medicine, Nanyang Technological University, Singapore, Singapore; 6 Department of Clinical Pharmacology, Medical University of Vienna, Vienna, Austria; 7 Center for Medical Physics and Biomedical Engineering, Medical University of Vienna, Vienna, Austria

## Abstract

We investigated the characteristics of the choriocapillaris flow voids using optical coherence tomography angiography (OCTA) in 85 patients (164 eyes) with hypertension (mean ± SD age, 56 ± 11 years; 45% women; 20% poorly controlled BP; 16% diabetes) who are without ocular diseases and determined possible correlations with systemic vascular risk factors. Data on 24-hour ambulatory blood pressure (BP), serum creatinine, and urine microalbumin/creatinine ratio (MCR) were collected. Estimated glomerular filtration rate (eGFR) was calculated based on CKD-EPI Creatinine Equation. OCTA imaging (6 × 6 mm scans; AngioVue) with quantitative microvascular analysis of the choriocapillaris was performed. Linear regression was used to investigate the association of systemic risk factors with area (percentage), size (pixels) and number of choriocapillaris flow voids. Diabetes (β = 0.33; 95% CI, 0.02 to 0.63) and daytime systolic BP (β = −0.13; 95% CI, −0.24 to −0.02) were associated with areas of flow voids. Age (β = 0.21; 95% CI, 0.06 to 0.36) and daytime diastolic BP (β = −0.18; 95% CI, −0.34 to −0.02) were associated with size of flow voids. Age (β = −21.21; 95% CI, −31.79 to −10.63) and nighttime diastolic BP (β = 13.89; 95% CI, 0.61 to 27.17) were associated with number of flow voids. Kidney parameters were not associated with any features of flow voids. In patients with hypertension, a higher BP appeared to increase blood flow in the choriocapillaris which needs to be considered when using the OCTA to study eye diseases in hypertensives.

## Introduction

Age-related macular degeneration (AMD) is the leading cause of central visual loss in the elderly population^1^. There is increasing evidence that the choroid plays a crucial pathophysiological role in the development of AMD^2^. Recent studies using optical coherence tomography (OCTA) showed that eyes with AMD tended to have less perfused choriocapillaris (e.g., larger areas of flow voids)^3–6^. This is in keeping with two longitudinal studies indicating the low choroidal blood flow as assessed with laser Doppler flowmetry, was associated with an increased risk for developing choroidal neovascularization in AMD^7,8^.

Systemic hypertension has been shown in various studies to be associated with AMD or choroidal neovascularization (CNV)^9–12^. This finding has suggested a role of systemic vascular abnormalities in the development and worsening of AMD. Studies correlating blood flow in the choroid and hypertension have yielded discordant conclusions thus far. Spaide *et al*. showed larger areas of flow voids in eyes of hypertensives compared to non-hypertensives when using OCTA^6^. Among the non-hypertensives, Polak *et al*. reported a small, but significant increase in choroidal blood flow in subjects with increasing BP^13^. It is uncertain whether the observed disparity between these studies were due to the influence of eye diseases instead of systemic disease (inclusion of AMD cases in Spaide’s^6^ vs eyes that are without any eye diseases in Polak’s^13^), different imaging technologies (i.e. OCTA vs laser interferometry and color Doppler imaging) or absence of BP measurements in Spaide’s^6^ or the inaccuracy of one-off office BP measurements in Polak’s^13^.

Furthermore, chronic kidney disease has also been shown to be associated with AMD^14^. The choroid and the kidney has the highest perfusion rate per gram tissue^15,16^. Therefore, systemic vascular influences can presumably affect both the choroid and kidney circulatory parameters. Previous study relied solely on the subjective-based questionnaire and reported no significant associations between history of kidney diseases and choroidal blood flow parameters using laser Doppler flowmetry^17^. The subjective nature of the questionnaire that was used to elucidate medical histories may be less reliable than the objective measurements of kidney function using biological data (serum creatinine, urine microalbumin/creatinine ratio (MCR), estimated glomerular filtration rate (eGFR).

Despite the widespread use of OCTA technology in studying AMD eyes^18^, there is limited understanding on the impact of systemic vascular risks on choriocapillaris flow in eyes without eye diseases. In an effort to resolve this issue, we investigated the effect of 24-hour ambulatory BP and renal parameters on flow characteristics of the choriocapillaris using OCTA in adults with treated systemic hypertension. We hypothesized that systemic hypertensive patients with higher levels of BP or poorer kidney function will have larger areas of flow voids that are also increased in size and reduced in number.

## Results

Table 1 shows the characteristics of the participants with systemic hypertension, as stratified by their BP levels. The mean ± SD age of participants was 56 ± 11 years and 45% were female (n = 39). In terms of their BP control, 18 (21%) were classified as having intensive BP control, 50 (59%) having standard BP control and 17 (20%) having poor BP control. Among the BP control groups, persons with intensively controlled BP who tended to be on angiotensin-converting enzyme inhibitors and angiotensin II receptor blockers had lower levels of urine MCR and larger areas of flow voids (P < 0.05 each).

**Table 1:** Clinical characteristics of treated hypertensive participants, stratified by blood pressure control.

	Intensive BP control	Standard BP control	Poor BP control	*P value
Number. of participants	18	50	17	
Number. of eyes	35	95	34	
Gender, female (%)	10 (56%)	22 (44%)	7 (41%)	0.637
Ethnicity, Chinese (%)	14 (78%)	43 (86%)	14 (82%)	0.870
Smoking Status, Never (%)	18 (100%)	45 (90%)	15 (88%)	0.575
Diabetes (%)	6 (33%)	5 (10%)	3 (18%)	0.082
Hyperlipidemia (%)	10 (56%)	23 (46%)	7 (41%)	0.677
Chronic Kidney Disease (%)	1 (6%)	3 (6%)	1 (6%)	1.000
Type of antihypertensive medications				
Beta blockers	5 (28%)	22 (44%)	4 (24%)	0.219
Calcium channel blockers	6 (33%)	28 (56%)	12 (71%)	0.080
Angiotensin-converting enzyme inhibitors and angiotensin II receptor blockers	16 (89%)	24 (48%)	7 (41%)	0.005
Others (diuretics, alpha 2 adrenergic agonist)	4 (22%)	5 (10%)	2 (12%)	0.410
Numbers of antihypertensive medications				
One	7 (39%)	29 (58%)	10 (59%)	0.620
Two	8 (44%)	13 (26%)	5 (29%)	
Three or more	3 (17%)	8 (16%)	2 (12%)	
**Mean** ± **SD/median (IQR)**				
Age, years	55 ± 7	57 ± 11	56 ± 11	0.834
Body mass index, kg/m^2^	27 (25–30)	26 (24–29)	25 (24–30)	0.645
Ambulatory BP, mmHg				
Systolic BP	112 ± 4	128 ± 5	146 ± 9	<0.001
Diastolic BP	72 ± 5	77 ± 7	85 ± 7	<0.001
Estimated GFR, mL/min/1.73 m^2^	94 (88–102)	91 (75–101)	95 (87–105)	0.191
Creatinine, µmol/L	67 (55–79)	77 (59–92)	73 (62–80)	0.159
Urine MCR, mg/g	0.9 (0–2.5)	0.6 (0–2.2)	2.3 (0.9–3.7)	0.022
**Choriocapillaries flow voids**				
Area of flow voids, %	17.2 (16.5–17.8)	16.8 (16.2–17.2)	16.7 (16.4–17.1)	0.017
Size of flow voids, pixels	10.8 ± 0.9	10.6 ± 0.8	10.9 ± 0.9	0.321
Number of flow voids	1199 ± 65	1191 ± 70	1176 ± 65	0.305

SD = standard deviation; IQR = interquartile range; BP = blood pressure; GFR = glomerular filtration rate; MCR = microalbumin-to-creatinine ratio.

Data are number (%) or mean ± SD or median (interquartile range), as appropriate.

*Test for differences between groups, based on analysis of variance (anova) for normally distributed continuous variables or kwallis for non-normally distributed continuous variables and with chi-square tests or Fisher’s exact test for categorical variables. Bold face indicates statistically significant P value.

**Figure 1:**
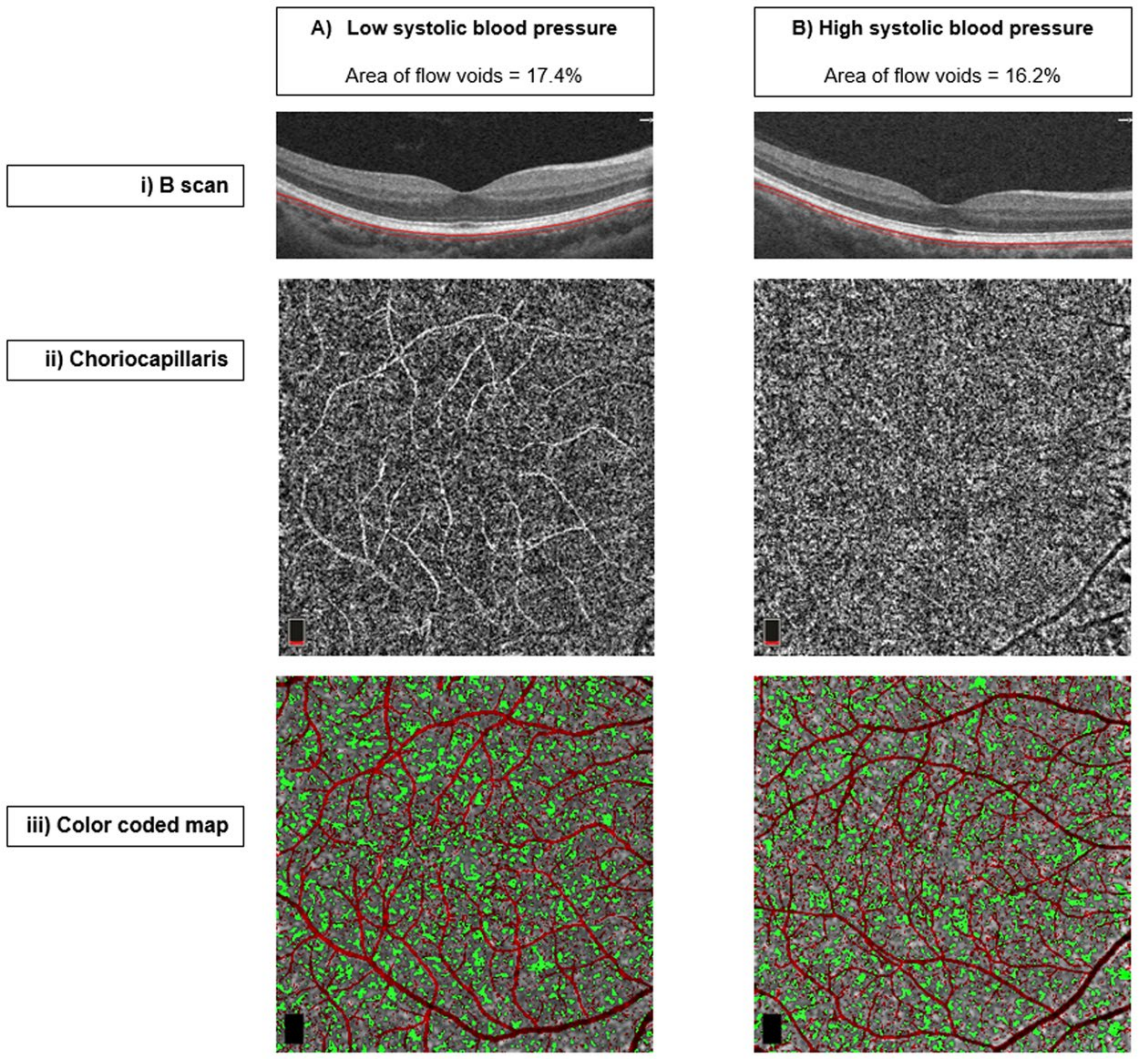
Area of choriocapillaris flow voids on optical coherence tomography angiography (OCTA) correlated significantly with ambulatory systolic blood pressure. (i) B scan of choriocapillaris. (ii) Choriocapillaris en face plexus. (iii) Color coded map of the flow voids (green) and superficial vascular plexus (red). Choriocapillaris flow voids map showing participant with (**A**) Low systolic blood pressure (larger area of flow voids) and (**B**) Low systolic blood pressure (smaller area of flow voids).

Associations between systemic factors and areas of flow voids are shown in Table 2. Univariate linear regression analysis showed that gender, diabetes, hypertensive medications (diuretics, alpha 2 adrenergic agonist), BP control, systolic BP, daytime systolic BP, diastolic BP and daytime diastolic BP were associated with areas of flow voids (all P < 0.10; Table 2). Age and kidney parameters were not significantly associated with areas of flow voids in the univariate model. In the multivariate-adjusted model, diabetes (β = 0.33; 95% CI, 0.02 to 0.63; P = 0.039), lower systolic BP (β = −0.11; 95% CI, −0.22 to −0.01; P = 0.046), and lower daytime systolic BP (β = −0.13; 95% CI, −0.24 to −0.02; P = 0.025; Table 2) were associated with larger areas of flow voids. Figure 1 further illustrates the relation of systolic BP and areas of flow voids showing that a person with lower systolic BP often had larger areas of flow voids compared to a person with higher systolic BP having smaller areas of flow voids. Age, gender, hypertensive medications (diuretics, alpha 2 adrenergic agonist), BP control, and diastolic BP were not significantly associated with areas of flow voids in the multivariate-adjusted model.

**Table 2:** Associations of systemic factors with area of flow voids (dependent variable) in hypertensive participants (n = 164 eyes).

	Univariate			Multivariate-adjusted		
	β	95% CI	P value	β	95% CI	P value
**Age, per 10 years**	0.05	−0.09 to 0.20	0.477	0.02	−0.12 to 0.17	0.738
**Gender**	−0.28	−0.56 to 0.08	0.056	−0.24	−0.51 to 0.03	0.084
**Diabetes**	0.36	−0.01 to 0.73	0.050	0.33	0.02 to 0.63	0.039
**Type of antihypertensive medications**						
Beta blockers	−0.01	−0.30 to 0.28	0.959	—	—	—
Calcium channel blockers	−0.11	−0.41 to 0.19	0.469	—	—	—
Angiotensin-converting enzyme inhibitors and angiotensin II receptor blockers	0.10	−0.19 to 0.38	0.502	—	—	—
Others (diuretics, alpha 2 adrenergic agonist)	0.32	−0.01 to 0.65	0.051	0.29	−0.03 to 0.62	0.072
**Numbers of antihypertensive medications**						
One		Reference				
Two	−0.06	−0.38 to 0.25	0.691	—	—	—
Three or more	0.18	−0.19 to 0.56	0.334	—	—	—
**BP control status**						
Intensive BP control	Reference			Reference		
Standard BP control	−0.40	−0.78 to −0.02	0.037	−0.29	−0.66 to 0.09	0.134
Poor BP control	−0.42	−0.87 to 0.03	0.067	−0.36	−0.78 to 0.06	0.093
**Systolic BP**						
Systolic BP, overall, per 10 mmHg	−0.13	−0.24 to −0.01	0.030	−0.11	−0.22 to −0.01	0.046
Systolic BP, day, per 10 mmHg	−0.15	−0.26 to −0.03	0.015	−0.13	−0.24 to −0.02	0.025
Systolic BP, night, per 10 mmHg	−0.05	−0.14 to 0.075	0.365	—	—	—
**Diastolic BP**						
Diastolic BP, overall, per 10 mmHg	−0.19	−0.37 to −0.02	0.032	−0.13	−0.31 to 0.06	0.173
Diastolic BP, day, per 10 mmHg	−0.20	−0.37 to −0.03	0.019	−0.15	−0.33 to 0.04	0.115
Diastolic BP, night, per 10 mmHg	−0.10	−0.26 to 0.07	0.238	—	—	—
**Kidney Parameters**						
Estimated GFR, mL/min/1.73 m^2^	0.04	−0.56 to 0.65	0.886	—	—	—
Creatinine	−0.39	−0.87 to 0.09	0.112	—	—	—
Urine MCR, mg/g	0.03	−0.12 to 0.18	0.709	—	—	—

CI = confidence interval; BP = blood pressure; GFR = glomerular filtration rate; MCR = microalbumin-to-creatinine ratio.

Multivariate model - adjusted for age, gender, diabetes, other types of BP medications, BP control and systolic BP.

Table 3 show the association between systemic factors and size of flow voids. Univariate linear regression analysis showed that age, diabetes, calcium channel blockers, diastolic BP and daytime diastolic BP were associated with size of flow voids (all P < 0.10; Table 3). BP control, systolic BP and kidney parameters were not associated with size of flow voids in the univariate model. In a multivariate model, older age (β = 0.21; 95% CI, 0.06 to 0.36; P = 0.006), calcium channel blockers (β = 0.31; 95% CI, 0.04 to 0.57; P = 0.024), lower diastolic BP (β = −0.19; 95% CI, −0.37 to −0.01; P = 0.034), and lower daytime diastolic BP (β = −0.18; 95% CI, −0.34 to −0.02; P = 0.032; Table 3) were associated with larger size of flow voids. Diabetes was not significantly associated with size of flow voids in the multivariate-adjusted model.

**Table 3:** Associations of systemic factors with size of flow voids (dependent variable) in hypertensive participants (n = 164 eyes).

	Univariate			Multivariate-adjusted		
	β	95% CI	P value	β	95% CI	P value
**Age, per 10 years**	0.27	0.11 to 0.43	0.001	0.21	0.06 to 0.36	0.006
**Gender**	−0.02	−0.33 to 0.29	0.894	0.07	−0.22 to 0.36	0.638
**Diabetes**	0.52	0.03 to 1.01	0.039	0.27	−0.17 to 0.72	0.226
**Type of antihypertensive medications**						
Beta blockers	−0.09	−0.41 to 0.24	0.593	—	—	—
Calcium channel blockers	0.29	−0.01 to 0.59	0.062	0.31	0.04 to 0.57	0.024
Angiotensin-converting enzyme inhibitors and angiotensin II receptor blockers	−0.02	−0.32 to 0.29	0.921	—	—	—
Others (diuretics, alpha 2 adrenergic agonist)	−0.05	−0.42 to 0.33	0.805	—	—	—
**Numbers of antihypertensive medications**						
One	Reference			Reference		
Two	0.07	−0.28 to 0.41	0.696	—	—	—
Three or more	0.11	−0.37 to 0.60	0.653	—	—	—
**BP control status**						
Intensive BP control	Reference			Reference		
Standard BP control	−0.15	−0.53 to 0.23	0.433	—	—	—
Poor BP control	0.11	−0.38 to 0.60	0.666	—	—	—
**Systolic BP**						
Systolic BP, overall, per 10 mmHg	−0.03	−0.15 to 0.09	0.634	—	—	—
Systolic BP, day, per 10 mmHg	−0.04	−0.15 to 0.08	0.534	—	—	—
Systolic BP, night, per 10 mmHg	0.00	−0.12 to 0.11	0.952	—	—	—
**Diastolic BP**						
Diastolic BP, overall, per 10 mmHg	−0.27	−0.45 to −0.08	0.004	−0.19	−0.37 to −0.01	0.034
Diastolic BP, day, per 10 mmHg	−0.27	−0.44 to −0.09	0.002	−0.18	−0.34 to −0.02	0.032
Diastolic BP, night, per 10 mmHg	−0.20	−0.40 to 0.01	0.056	−0.17	−0.36 to 0.02	0.082
**Kidney Parameters**						
Estimated GFR, mL/min/1.73 m^2^	−0.46	−1.26 to 0.34	0.262	—	—	—
Creatinine	−0.15	−0.71 to 0.42	0.604	—	—	—
Urine MCR, mg/g	0.12	−0.04 to 0.29	0.138	—	—	—

CI = confidence interval; BP = blood pressure; GFR = glomerular filtration rate; MCR = microalbumin-to-creatinine ratio.

Multivariate model - adjusted for age, gender, diabetes, calcium channel blockers and diastolic BP.

The association between systemic factors and numbers of flow voids is shown in Table 4. Univariate linear regression analysis showed that age, calcium channel blockers, diastolic BP, daytime diastolic BP and nighttime diastolic BP were associated with numbers of flow voids (all P < 0.10; Table 4). Diabetes, BP control, systolic BP and kidney parameters were not associated with numbers of flow voids in the univariate model. In a multivariate model, younger age (β = −21.21; 95% CI, −31.79 to −10.63; P < 0.001), absence of calcium channel blockers (β = −24.48; 95% CI, −44.43 to −4.53; P = 0.016), and higher nighttime diastolic BP (β = 13.89; 95% CI, 0.61 to 27.17; P = 0.040; Table 4) were significantly associated with greater number of flow voids.

**Table 4:** Associations of systemic factors with number of flow voids (dependent variable) in hypertensive participants (n = 164 eyes).

	Univariate			Multivariate-adjusted		
	β	95% CI	P value	β	95% CI	P value
**Age, per 10 years**	−22.82	−34.51 to −11.13	<0.001	−21.21	−31.79 to −10.63	<0.001
**Gender**	−8.25	−31.67 to 15.17	0.490	−15.08	−0.22 to 0.36	0.164
**Diabetes**	−20.42	57.86 to 17.03	0.285	−3.00	−35.32 to 29.33	0.856
**Type of antihypertensive medications**						
Beta blockers	9.30	−17.66 to 36.26	0.499	—	—	—
Calcium channel blockers	−19.12	−41.53 to 3.30	0.095	−24.48	−44.43 to −4.53	0.016
Angiotensin-converting enzyme inhibitors and angiotensin II receptor blockers	4.02	−18.86 to 26.90	0.731	—	—	—
Others (diuretics, alpha 2 adrenergic agonist)	7.99	−21.24 to 37.23	0.592	—	—	—
**Numbers of antihypertensive medications**						
One	Reference			Reference		
Two	−6.67	−32.27 to 18.92	0.609	—	—	—
Three or more	3.14	−40.48 to 46.76	0.653	—	—	—
**BP control status**						
Intensive BP control	Reference			Reference		
Standard BP control	−5.34	−32.54 to 21.87	0.701	—	—	—
Poor BP control	−24.48	−55.62 to 6.66	0.123	—	—	—
**Systolic BP**						
Systolic BP, overall, per 10 mmHg	−1.46	−10.85 to 7.94	0.761	—	—	—
Systolic BP, day, per 10 mmHg	−1.79	−10.69 to 7.10	0.693	—	—	—
Systolic BP, night, per 10 mmHg	−0.05	−8.56 to 8.47	0.992	—	—	—
**Diastolic BP**						
Diastolic BP, overall, per 10 mmHg	16.40	3.71 to 29.10	0.011	12.16	−0.03 to 24.36	0.051
Diastolic BP, day, per 10 mmHg	15.19	28.62 to 27.53	0.016	10.13	−1.35 to 21.61	0.084
Diastolic BP, night, per 10 mmHg	15.15	1.27 to 29.03	0.032	13.89	0.61 to 27.17	0.040
**Kidney Parameters**						
Estimated GFR, mL/min/1.73 m^2^	49.16	−7.53 to 105.86	0.089	—	—	—
Creatinine	−4.39	−45.13 to 36.35	0.833	—	—	—
Urine MCR, mg/g	−8.56	−23.87 to 6.76	0.274	—	—	—

CI = confidence interval; BP = blood pressure; GFR = glomerular filtration rate; MCR = microalbumin-to-creatinine ratio.

Multivariate model - adjusted for age, gender, diabetes, calcium channel blockers and diastolic BP.

**Figure 2:**
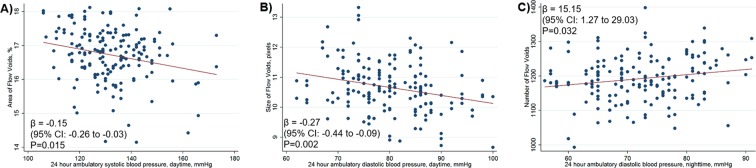
Scatterplot showing negative correlations of (**A**) area of choriocapillaris flow voids with 24-hour ambulatory daytime systolic blood pressure and (**B**) size of choriocapillaris flow voids with 24-hour ambulatory daytime diastolic blood pressure.

Figure 2 further illustrates the association observed between BP with varying features (by area, size and number) of choriocapillaris flow voids. In patients with hypertension, a higher BP appeared to have smaller area of flow voids (β = −0.15; 95% CI, −0.26 to −0.03; P = 0.015; Fig. 2A), that are also reduced in size (β = −0.27; 95% CI, −0.44 to −0.09; P = 0.002; Fig. 2B) and greater in numbers (β = 15.15; 95% CI, 1.27 to 29.03; P = 0.032; Fig. 2B).

## Discussion

Certain features of choriocapillaris flow voids were significantly correlated with 24-hour ambulatory BP, presence of diabetes and use of calcium channel blockers in systemic hypertensive patients. Our results suggest increased choroidal blood circulation in patients with uncontrolled hypertension, which supports the potential application of using OCTA technology to detect alterations of choroidal perfusion in systemic disease.

The present data advance our knowledge on the impact of BP on the choroidal blood flow in several ways. First, systemic hypertensive patients having higher BP are more likely to have smaller area of flow voids. Also, these flow voids are generally smaller in average size and more numerous, compared to those with lower BP. This suggests that in hypertensive patients, the increasing levels of BP would lead to an increase in blood flow within the choriocapillaris. This finding is contrary to that reported by Spaide *et al*.^6^. He studied the flow characteristics of the choriocapillaris in 80 patients aged 24–99 years old and showed larger area of flow void in individuals with systemic hypertension^6^. In his study, there was no mention on the BP levels among the controls and hypertension group, which can result in biasness. Instead, our study is in good agreement with Polak’s previous published work. She investigated choroidal flow using laser interferometry and color Doppler imaging in 318 middle-aged male volunteers who did not receive any systemic medication and found a significant correlation between higher levels of BP and an increase in choroidal blood flow^13^.

The impact of BP was in the opposition direction for the retinal vessels. In the same cohort of patients with systemic hypertension, we observed a sparser retinal capillary density with increasing systolic BP, potentially associated with hypertension-induced retinal capillary dropout^19^. Previous studies on the association between retinal blood flow and different techniques to assess retinal blood flow did not show an association between BP and retinal perfusion^20,21^. Autoregulation is known as the ability of a vascular bed to keep blood flow relatively constant despite alterations in perfusion pressure. The choroid has long been assumed to be a passive vascular bed, that is, one that does not show autoregulation. Indeed, early studies have indicated if perfusion pressure is increased to the choroid, there is a linear increase in blood flow^22^. Contrary to this notion, the choroid has shown some regulatory compensation response when the ocular perfusion pressure was experimentally modified in later studies^23–25^. In such experiments^23–25^, the effect of acute changes in perfusion pressure is studied and results can not necessarily be extrapolated to systemic hypertension, which represents the persistent elevation of systemic BP. Our results indicate that choroidal flow was greater in those with higher BP. The slope of the regression line is, however, flat indicating a non-linear relation between BP and choroidal flow. Therefore, the data support the concept of choroidal autoregulation, but the choroidal vasculature appears to have lesser autoregulatory capacity than the retinal vasculature^26^.

The mechanism underlying the impact of hypertension on the ocular microvasculature is unclear based on the present data. The posterior tissues of the eye are supplied by two vascular supply with distinct properties^26^. For the retinal vasculature, it has been shown that chronic systemic hypertension is associated with narrower blood vessels^27–29^. This indicates that an elevated vascular resistance is associated with a dysfunctional autoregulation of the retinal vasculature^30^. In the choroid, however, no study has yet shown that hypertension is associated with arteriolar vasoconstriction. Whether an increase in choroidal perfusion is protective against AMD remains unclear. It is plausible that a higher blood flow in the choroid may prevent hypoxia in the outer retina^31^. On the contrary, choroidal overperfusion may cause oxidative stress to the outer retina, which has been postulate to contribute to the development of AMD^32^.

The presence of microvascular changes in the eye, such as an increased in size and lesser in numbers of flow voids using OCTA, was independently associated with older age. This is in keeping with what Spaide had already published on age and flow voids^6^. Using statistical modeling, he demonstrated that the appearance of choriocapillaris flow voids followed a power-law distribution, initially with many small flow voids and progressively fewer larger flow voids. This is in keeping with a variety of other studies using magnetic resonance imaging^33^, laser Doppler flowmetry^34^, or laser interferometry^35^. Whether this age-related decline in choroidal perfusion is due to reduced metabolic demand or a risk factor for eye disease is unknown.

We also saw that individuals taking calcium channel blockers had choriocapillaris flow voids that were larger in average size and lesser in numbers. Calcium channels are known to regulate smooth muscles, which may also impact the regulation of blood flow in the choroid. Calcium channel blockers relax and widen blood vessels and trigger the release of nitric oxide^36^. Nifedipine, an L-type calcium channel blocker, altered the regulation of choroidal blood flow during isometric exercise in 15 healthy men^37^. Various studies using animal models, have also indicated that calcium channel blockers may increase ocular blood flow^38–40^. Verapamil, however, reversed the downward shift induced by nitric oxide synthase inhibition in the choroidal pressure/flow relationship in the rabbit^41^. The relatively small sample size of persons taking different types of calcium channel blockers prevented post-hoc analysis of specific calcium channel blockers on choroidal flow voids.

What is the immediate clinical relevance of our study? Systemic parameters such as BP levels, diabetes status and hypertensive medications can impact the choriocapillaris and should be considered by future studies. Current studies have mostly defined their controls as individuals without overt eye diseases^3–5,42^, with the exception of one study which further excluded participants if they had diabetes or hypertension^43^. When using the OCTA to examine the role of altered choroidal microvasculature in eye diseases, clinicians should account for patients’ systemic health status and medication information, which can thereby lead to a more valid conclusion.

One of the major strength of the current study is the usage of reliable and objective clinical tests, which include 24-hr ambulatory BP monitoring, urine MCR and eGFR. There are several limitations that need to be emphasized. First, the flow voids that we have visualized within the choriocapillaris could also be a result of an extremely slow flow rate. Currently, OCTA visualizes blood flow within the vessels by detecting motion contrast from blood flow. A vessel having very low blood flow rate, one that is below the detection threshold of the OCTA device, will not be elucidated^44^. For this reason, the term “flow void” refers to the signal loss occurring within the flowing blood. Second, even though we report smaller areas of flow voids that are reduced in size and greater in numbers among those with higher BP, this was only a cross-sectional study. OCTA was recently made available in the clinic and follow-up study is in progress. Third, smoking is known to reduce blood flow of the choriocapillaris within the macular region by the acute effects of nicotine in cigarettes, as evaluated by OCTA^45^. Moreover, abnormal choroidal blood flow regulation in response to modifications in ocular perfusion pressure was seen in smokers^46^. However, we did not assess this relation of smoking on choriocapillaris flow due to a paucity of smokers. Fourth, the lack of ocular parameters such as intraocular pressure^47^ and axial length^48^ may confound the measurements of flow voids as these ocular factors are known to have an effect on flow voids. Last, we did not calculate the repeatability of our measurements. However, a previous publication reported the repeatability of flow voids measurements to be excellent^3^.

In conclusion, we have demonstrated the impact of ambulatory BP on choriocapillaris flow voids in adults with treated systemic hypertension. Choriocapillaris flow voids measured using the OCTA may mirror systemic microvascular dysfunction due to systemic hypertension. This suggests the possible use of the OCTA as a device to detect systemic microvascular dysfunction. Future studies are needed to comprehend the impact that eye diseases such as AMD have on the choroidal vasculature.

## Methods

### Study Participants

We conducted a prospectively planned observational cross-sectional study including 164 eyes from 85 from participants with essential hypertension enrolled in the Response of the Myocardium to Hypertrophic Conditions in the Adult Population (REMODEL; Response of the myocardium to hypertrophic conditions in the adult population; NCT02670031)^49,50^. Briefly, Asians with essential hypertension on antihypertensive medications, aged 18 years and older, were recruited from a tertiary cardiac centre and primary care clinics in Singapore, from January 2017 to February 2018. Participants with secondary causes of hypertension, any on-going unstable medical conditions, previously diagnosed significant coronary artery disease (defined as previous myocardial infarction, more than 70% coronary stenosis on invasive coronary angiography or positive cardiac stress tests), strokes, atrial fibrillation and women who are pregnant or breast feeding were excluded from the study^49^.

Study was approved by the SingHealth Centralized Institutional Review Board, conducted in accordance to the Declaration of Helsinki, where written informed consents were obtained from participants.

### Examination procedures

Detailed interviewer-administered questionnaire was used to collect demographic data, lifestyle risk factors, medical history and medication use^51^. Ethnicities were set by the Singapore census^52^. 24-hour ambulatory BP (systolic blood pressure, SBP and diastolic blood pressure, DBP) were measured in all participants. In the current analysis, daytime ambulatory BP was measured at intervals of every 20 minutes from 6 am to 10 pm and nighttime ambulatory BP was measured at intervals of every 30 minutes from 10 pm to 6 am. Hypertensive patients were stratified into three groups based on the Systolic Blood Pressure Intervention Trial (SPRINT)^53^: Intensive BP control defined as systolic BP <120 mmHg, standard BP control as systolic BP 120–139 mmHg and poor BP control as systolic BP ≥140 mmHg. Diabetes was defined based on self-reported physician diagnosed diabetes or glucose-lowering medications. Hyperlipidemia was defined based on clinical history of elevated cholesterol or lipid-lowering medications. Participants’ height was measured using a wall-mounted measuring tape and weight was measured using a digital scale (SECA, model 782 2321009, Germany)^54^. Body mass index (BMI) was calculated as body weight (in kilograms) divided by body height (in meters) squared. Smoking status was defined as those never smoked, current smokers and past smokers.

Blood and mid-stream urine samples were collected for analysis of serum creatinine and urine microalbumin/creatinine ratio (MCR). Bio-specimens were processed in an accredited laboratory at the Singapore General Hospital. eGFR (in mL/min/1.73 m^2^) was calculated from plasma creatinine using the recently developed Chronic Kidney Disease Epidemiology Collaboration (CKD-EPI) equation^55^. MCR was measured using immunoassay. Normal MCR range was 0.2 to 3.3 mg/mmol creatinine whilst values >33.9 mg/mmol creatinine implied clinical albuminuria.

### Ocular examinations

Participants underwent a questionnaire regarding their ocular history (e.g. intraocular surgery or glaucoma). To mitigate the risk of angle-closure glaucoma, we performed a pre-dilation check. Intraocular pressure was measured using noncontact tonometry (Auto Non-Contact Tonometer, NT-3000; Nidek, Gamagori, Japan)^19^. Persons having an intraocular pressure reading of less than 21 mmHg went on to receive pupillary dilation and imaging scans. Conversely, those having an intraocular pressure reading of 21 mmHg and above exited from the study. Fundus photography and OCTA were performed approximately 30 minutes after topical instillation of 2 drops of 1% tropicamide, given 5 minutes apart. Fundus photography was performed using a retinal camera (Canon CR-DGi with a 10-DSLR back; Canon, Tokyo, Japan) to ascertain the presence of eye disease^56^. Patients with eye diseases (e.g. glaucoma, vascular or nonvascular retinopathies, and age-related macular degeneration) were excluded from the study^56^.

### Optical coherence tomography angiography

The OCTA imaging system provides a non-invasive method for visualizing the choriocapillaris (AngioVue; Optovue, Inc., Fremont, California USA)^57^. The AngioVue OCTA employs the split-spectrum amplitude decorrelation angiography (SSADA) algorithm to acquire flow signal^58^ and allows a high-resolution 3-dimensional visualization of perfused vasculature^57,59^. The device has an A-scan rate of 70,000 scans per second and 2 successive B-scans were taken at the same location. Each imaging cube consisted of 2 repeated volumes (304 B-scans × 304 A-scans). For this study, we used choriocapillaris flow measurements within the macula, in scans with a 6.0 × 6.0 mm^2^ field of view centered on the fovea^59^. Each scan was automatically segmented by the AngioVue software (version 2016.2.0.35) so as to visualize the superficial vascular plexus and choriocapillaris.

### Method for measuring choriocapillaris flow voids

**Figure 3:**
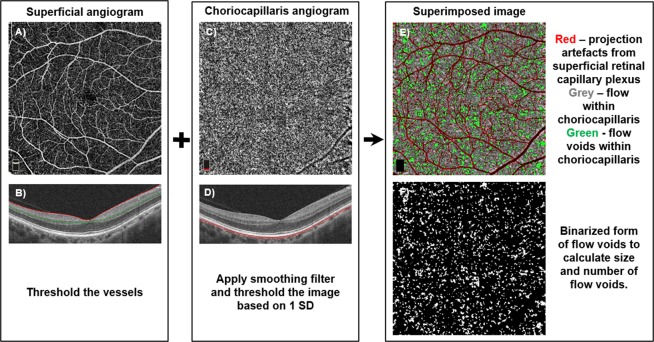
Algorithm for artifact removal and calculation of features of choriocapillaris flow voids.

We calculated the area of choriocapillaris flow voids based on an image-processing algorithm as described previously by Nesper *et al*. (Fig. 3)^3^. Briefly, the *en face* angiograms of the (1) superficial retinal capillary plexus (Fig. 3A) (3 μm below the inner limiting membrane to 16 μm below inner plexiform layer; Fig. 3B) and (2) choriocapillaris (Fig. 3C) (31 μm to 59 μm below the RPE reference; Fig. 3D) were segmented from the OCTA device^3^. The images of the superficial capillary plexus and the choriocapillaris slabs were then imported into MATLAB (MathWorks, Inc., Natick, Massachusetts)^3^.

Superficial retinal capillary plexus left noticeable projection artefacts on the choriocapillaris which may confound the calculation of flow voids^3^. To remove the shadow artifacts on the choriocapillaris, we thresholded the superficial retinal vascular plexus image to create a binary mask (red color; Fig. 3E). We then overlaid the mask on the choriocapillaris image, which helped us to identify the artifacts on the choriocapillaris image. Regions in the choriocapillaris that were caused by projection artifacts of the superficial retinal vascular plexus were excluded in the calculation of the percentage of flow voids.

To identify the flow voids in the choriocapillaris image, we first applied a Gaussian smoothing filter (σ = 20 μm) to reduce the speckle noise in the choriocapillaris image and then thresholded the choriocapillaris image, as described previously by Zhang *et al*.^42^:

$${I}_{mean}-\,\eta \cdot SD$$

*I*_*mean*_ represents the average pixel intensity of the choriocapillaris image while 𝜂 denotes a positive number that was multiplied against the standard deviation of the pixel intensity of the choriocapillaris image. A value of 1 SD was used because Zhang *et al*. showed that it provided the best repeatability^42^. Pixels that fell below this threshold were considered as flow voids (green color; Fig. 3E).

The area of choriocapillaris flow voids was defined as a percentage between the region that is absent from flow and the total scanned region, as follows (Fig. 3E)^42^:

$$\frac{area\,of\,flow\,voids}{total\,area\,centred\,at\,fovea}\times 100 \% $$

The size and number of flow voids were calculated from a binarized image of the choriocapillaris flow voids (Fig. 3F). The binarized image was imported into ImageJ software (National Institutes of Health, Bethesda, MD; available at https://imagej.nih.gov/ij/) for analysis^5^. The image was threshold to select the flow voids and the “Distribution” command was then applied with the parameter set as “Area” to obtain the average size of the flow voids^60^. The “Analyze Particles” command was employed to count the number of flow voids present.

A trained grader masked to the participants’ characteristics reviewed the quality of all OCTA scans. Poor quality scans were excluded from the analysis if one of the following criteria were met: (1) poor clarity images; (2) local weak signal caused by artifacts such as floaters; (3) residual motion artifacts visible as irregular vessel patterns on the *en face* angiogram and (4) scans with segmentation failure^59^.

**Figure 4:**
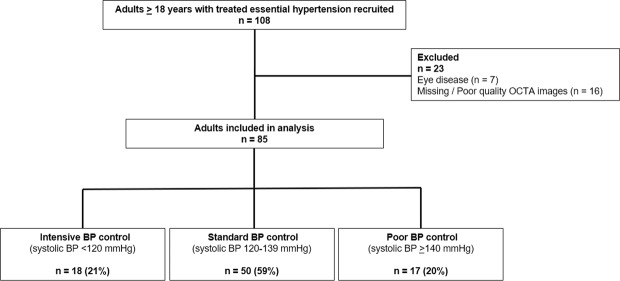
Identification of study participants. Of the 85 participants with systemic hypertension, 17 (20%) had poorly controlled blood pressure.

Of the 108 participants, we excluded participants with eye diseases (n = 7) and missing or poor quality OCTA images (n = 16), leaving 85 participants for analysis (Fig. 4). Comparisons between included and excluded participants revealed no differences in terms of their age (P = 0.09), systolic BP (P = 0.24) and diastolic BP (P = 0.56).

### Statistical analyses

Primary outcomes were features (by area, size and number) of choriocapillaris flow voids. The normality of the distribution of the continuous variables was assessed using Shapiro-Wilk test. To compare continuous variables among groups, a 1-way analysis of variance (ANOVA) was performed for normally distributed variables whereas Kruskal-Wallis test was used for non-normally distributed variables. Continuous variables that were normally distributed are presented as mean ± standard deviation (SD) whereas non-normally distributed variables are presented as median (interquartile range [IQR]). Chi-square test or Fisher’s exact test were used for categorical variables. There was a strong correlation between the right and left eyes for area (Pearson correlation coefficient, r = 0.65; P < 0.001) and size (r = 0.65; P < 0.001) of flow voids. However, the correlation was weaker for number of flow voids (r = 0.49; P < 0.001). Hence, generalized estimating equations were used to account for inter-eye correlation since data from both eyes were used^61,62^. Associations between systemic factors (independent variables) with features of choriocapillaris flow voids (dependent variable) were assessed using univariate and multivariate linear regression models with generalized estimating equations^63^. In addition to biologically plausible factors such as age, gender, and diabetes, we also used the univariate analysis as an initial step to select covariates for further consideration in the multivariate regression, factors with P < 0.10 in the univariate model were included in the multivariate model^63^. Data were analyzed with statistical software (STATA, version 13.1; StataCorp LP).

## Data Availability

The datasets generated during and/or analyzed during the current study are not publicly available due to the terms of consent to which the participants agreed but are available from the corresponding author on reasonable request.
